# Efficacy and safety of Kumpe access catheter for pre-percutaneous nephrolithotomy renal access in modified supine percutaneous nephrolithotomy

**DOI:** 10.1186/s12894-023-01227-4

**Published:** 2023-06-15

**Authors:** Jun-Koo Kang, Sang Hee Lee, Seok-Gi Kim, Ju-Bin Kim, Jeong-Yeon Lee, Seon-Yeon Ha, Chan-Geun Ha, Soon-Ho Hong, Jae-Wook Chung, Yun-Sok Ha, Jun Nyung Lee, So Young Chun, Bum Soo Kim

**Affiliations:** 1 Department of Urology, Kyungpook National University Hospital, 130 Dongdeok-Ro, Jung-Gu, Daegu, 41944 South Korea; 2 Department of Medical Science, Kyungpook National University, Daegu, South Korea; 3 Department of Urology, School of Medicine, Kyungpook National University, Daegu, South Korea; 4 BioMedical Research Institute, Kyungpook National University Hospital, Daegu, South Korea

**Keywords:** Kidney calculi, Nephrolithotomy, Percutaneous, Radiation exposure

## Abstract

**Introduction:**

Traditionally, a pigtail catheter (PCN) is placed for preoperative renal access before performing percutaneous nephrolithotomy (PCNL). However, PCN can hamper the passage of the guidewire to the ureter, due to which, access tract can be lost. Therefore, Kumpe Access Catheter (KMP) has been proposed for preoperative renal access before PCNL. In this study, we analyzed the efficacy and safety of KMP for surgical outcomes in modified supine PCNL compared to those in PCN.

**Materials and methods:**

From July 2017 to December 2020, 232 patients underwent modified supine PCNL at a single tertiary center, of which 151 patients were enrolled in this study after excluding patients who underwent bilateral surgery, multiple punctures, or combined operations. Enrolled patients were divided into two groups according to the type of pre-PCNL nephrostomy catheter used: PCN versus KMP. A pre-PCNL nephrostomy catheter was selected based on the radiologist’s preference. A single surgeon performed all PCNL procedures. Patient characteristics and surgical outcomes, including stone-free rate, operation time, radiation exposure time (RET), and complications, were compared between the two groups.

**Results:**

Of the 151 patients, 53 underwent PCN placement, and 98 underwent KMP placement for pre-PCNL nephrostomy. Patient baseline characteristics were comparable between the two groups, except for the renal stone type and multiplicity. The operation time, stone-free rate, and complication rate were not significantly different between the two groups; however, RET was significantly shorter in the KMP group.

**Conclusion:**

The surgical outcomes of KMP placement were comparable to those of PCN and showed shorter RET during modified supine PCNL. Based on our results, we recommend KMP placement for pre-PCNL nephrostomy, particularly for reducing RET during supine PCNL.

## Introduction

Urolithiasis is one of the most common diseases, with a high incidence and prevalence among three significant urologic diseases [1]. The treatment of urolithiasis is a complex process that requires the appropriate application of treatment methods with various levels of surgical difficulty in consideration of the nature and size of the stone, different anatomical characteristics and variations, etc. [2]. Percutaneous nephrolithotomy (PCNL), a representative surgery for removing kidney stones, is considered the first-line therapy for large kidney stones. Since Fernström and Johansson first performed PCNL in 1976 [3], there has been a significant development in surgical techniques and perioperative methods, such as patient positioning, renal access, tract dilation technique, lithotriptor, disintegration, and exit strategy [4].

PCNL is traditionally performed in a prone position. However, this has some limitations, such as the need to change patient’s position for ureteral catheter placement, higher risk of cardiopulmonary complications, and difficulty in using a simultaneous retrograde approach. Thus, to overcome these limitations, Valdivia et al. [5] introduced a supine position PCNL in 1987. This position reduces the risk of irrigation fluid and urine reabsorption because a lower pressure can be maintained in the renal pelvis than that in the prone PCNL. In addition, a simultaneous retrograde ureteroscopic approach can be used as it does not require changing patient’s position during surgery. With these advantages, supine PCNL has gained popularity. However, several disadvantages, including restricted dilation of the renal pelvis during surgery, relatively longer access tract, more challenging upper pole puncture, and more mobile kidney during tract dilation [6].

Renal access is one of the most important steps for a safe and successful PCNL. It can be achieved preoperatively by radiologists or intraoperatively by urologists depending on the surgeon’s preferences. At our institute, a radiologist usually makes renal access the day before surgery due to day before preoperative renal access shorten operation time and it can reduce the rate and severity of postoperative infection and significantly decrease the likelihood of post-PCNL sepsis [7, 8]. Traditionally, percutaneous nephrostomy (PCN) using a pigtail catheter is preferred for pre-PCNL renal access. However, it can hamper the passage of the guidewire to the ureter, eventually causing loss of access tract, especially during supine PCNL. Kumpe Access Catheter (KMP) has been recently used at our institute for easier guidewire passage to the ureter during tract dilation for pre-PCNL nephrostomy. In this study, we aimed to analyze the efficacy and safety of using KMP in pre-PCNL nephrostomy for the surgical outcomes of PCNL and compare them with those of PCN.

## Materials and methods

### Research ethics

This study was conducted using medical records and was approved by the Institutional Review Board of Kyungpook National University Hospital (approval number: KNUH 2022-09-037). The study was conducted in compliance with the principles of the Declaration of Helsinki. As this was a retrospective study, the requirement for informed consent was waived. To protect patient privacy, patient identifying information obtained from the computerized medical records was used as an encrypted Excel file.

### Research group setting

**Fig. 1:**
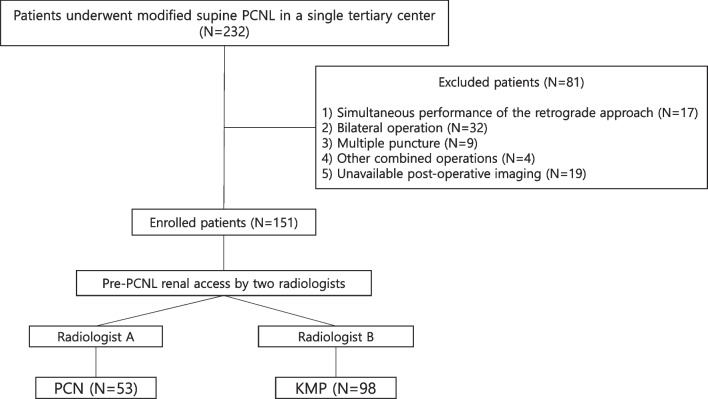
Basic schema of the study group information

Total 232 consecutive patients who underwent PCNL in a modified supine position between July 2017 and December 2020 at a single tertiary hospital were retrospectively reviewed. The exclusion criteria were: (1) simultaneous performance of the retrograde approach, (2) bilateral operation, (3) multiple punctures, (4) other combined operations, and (5) unavailable post-operative imaging. Finally, 151 patients were enrolled, of which 53 underwent PCN and 98 underwent KMP placement as pre-PCNL nephrostomy catheters (Fig. 1).

### Surgical procedure

**Fig. 2:**
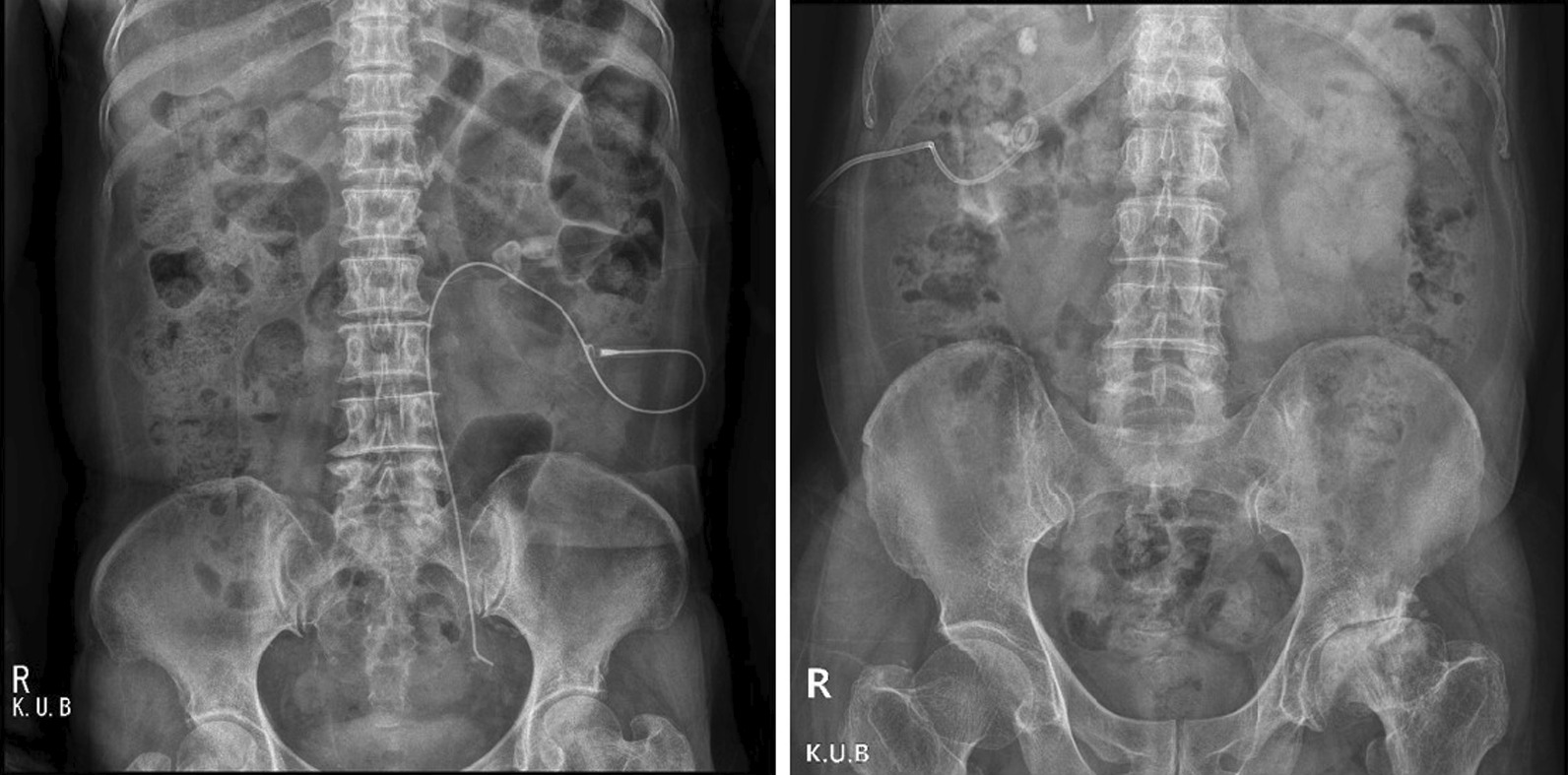
KMP and PCN catheter

On the day before surgery, pre-PCNL renal access was performed by two radiologists under ultrasound and fluoroscopy guidance. An 8.5 F pigtail catheter (PCN) or a 5 F KMP catheter was used based on the preference of each radiologist (Fig. 2). Among the two radiologists, the older one preferred traditional PCN indwelling, and the younger radiologist preferred KMP catheter indwelling. Renal calyx was punctured guided by ultrasonography. After making a puncture using the Chiba needle in the collecting system and confirming urine output through the needle, pyelography was performed to adjust the depth of needle tip. Then, a hairy guidewire was introduced into the renal pelvis and fascial dilator was followed. Through the fascial dilator, a 0.035 inch hydrophilic guidewire was introduced, and a pre-PCNL catheter was placed.

All PCNL surgeries were performed by a single surgeon in a Galdakao-modified supine Valdivia (GMSV) position. Subsequently, a 0.5–1.0 cm incision was expanded from the percutaneous nephrostomy tract, and a hydrophilic guidewire was passed through the pre-PCNL nephrostomy catheter.

Preoperative renal access and nephrostomy catheter placement are performed with the patient in the prone position. During supine PCNL, if the patient is tilted to the side during surgery, sufficient space cannot be secured around the puncture site, and tract dilators cannot easily pass through because of the hypermobile kidney. Therefore for tract dilation, if the guidewire, which passed through the nephrostomy catheter, smoothly passed to the ureter, the distal end of the hydrophilic guidewire was advanced into the bladder and extracted through the urethra using a cystoscope with foreign body forceps. Then, the proximal and distal ends of the guidewire were contralaterally pulled with tension for the easy placement of a fascia-cutting needle and nephrostomy balloon catheter [9]. However, if the guidewire did not pass through the ureter due to the presence of staghorn stone, impacted stone, or anatomical variance, a nephrostomy tract was made after coiling the distal end of the guidewire inside the renal pelvis or calyx. If the first guidewire was successfully placed, the pre-PCNL nephrostomy catheter was removed, a dual-lumen catheter was antegradely placed, and the second hydrophilic guidewire was antegradely introduced for safety. The nephrostomy tract was dilated using a nephrostomy balloon catheter. Then, an Amplatz access sheath was placed over the nephrostomy balloon catheter, and the UPJ occlusion balloon catheter was placed in a retrograde direction overriding the working guidewire, which was used for tract dilation.

In both the groups, a rigid nephroscope was introduced, and lithotripsy was performed using a pneumatic and ultrasonic lithotripter; in the case of mini-PCNL, a holmium laser was used for lithotripsy. Depending on the bleeding status of the nephrostomy tract, remnant stones, and the presence of collecting system damage, placement of a nephrostomy catheter, double-J stent, or tubeless/stentless was determined [10].

### Definition

Age, sex, body mass index (BMI), stone type, stone size, stone location, number of stones, puncture location, and Amplatz sheath size were compared between the two groups. When stones covered more than 80% or less than 80% of the collecting system, they were classified as complete and partial staghorn stones, respectively. The number of stones was considered either single or multiple stones, in case of two or more stones. Parenchymal thinning confirmed the presence of a thickness difference of at least 3 mm compared with the unaffected side, and caliectasis confirmed whether the calyx was dilated with hydronephrosis.

After surgery, the stone-free rate (SFR), operation time, complication rate, intraoperative radiation exposure time (RET), hospital stay, and mean hemoglobin drop were compared between both the groups. Criteria for stone-free status was based on non-contrast CT scans with 3 mm cuts at post-operative 1 month. Stone-free status was subclassified into three grades: Grade A (no residual stones), Grade B (residual stones less than 2 mm) and Grade C (residual stones between 2 and 4 mm). The incidence of complications was confirmed through follow-up. The three most common complication types were vascular embolism, transfusion, and febrile urinary tract infection (UTI).

### Statistical analysis

The data obtained for the patients in the two groups (KMP and PCN) were statistically analyzed. To compare the variables between the two groups, Student’s *t*-test was performed on continuous variables, and a chi-square test was performed on categorical variables. Multivariate logistic regression analysis was performed to identify the predictive variables for radiation exposure time. Results with *p* < 0.05 were considered statistically significant. Statistical analyses were performed using SPSS for Windows, version 23 (IBM Corp., Armonk, NY, USA).

## Results

Table 1 shows the baseline characteristics of the patients in the PCN and KMP groups. The overall mean age was 58.81 ± 13.79 years; 70.2% participants were male and 29.8%, participants were female. The mean BMI was 25.29 ± 3.30, which placed patients in the WHO Asian standard overweight group. The overall mean kidney stone size was 25.71 ± 10.75 mm. The proportions of kidney stones were 35.8% on the right side and 64.2% on the left side. Among the kidney stones, 14.6% were complete staghorn and 44.4% partial staghorn types; 55.6% cases showed multiplicity, and caliectasis and parenchymal thinning were observed in 55.0% and 31.8% of the cases, respectively. Moreover, in 89.4% of cases, the puncture was performed in the lower calyx.

**Table 1:** Basic characteristic of study groups that underwent PCNL in the modified supine position

Variable	PCN (N = 53)	KMP (n = 98)	*P* value
Age (year)	58.25 ± 14.54	59.12 ± 13.37	0.71
Gender			0.939
Male	37 (69.8)	69 (70.4)	
Female	16 (30.2)	29 (29.6)	
BMI	25.2 ± 3.20	25.34 ± 3.35	0.814
Laterality			0.707
Right	18 (34.0)	36 (36.7)	
Left	35 (66.0)	62 (63.3)	
Renal stone type			0.012
Complete staghorn	4 (7.5)	18 (18.4)	
Partial staghorn	19 (35.8)	48 (49.0)	
Others	30 (56.6)	32 (32.7)	
Renal stone size (mm)	23.59 ± 9.53	26.85 ± 11.35	0.063
Caliectasis	33 (62.3)	50 (51.0)	0.185
Parenchymal thinning	16 (30.2)	32 (32.7)	0.756
Multiplicity of renal stone			0.025
Single	17 (32.1)	50 (51.0)	
Multiple	36 (67.9)	48 (49.0)	
Tract size			0.11
30 Fr	41 (77.4)	28 (28.6)	
24 Fr	0 (0)	6 (6.1)	
18 Fr	12 (22.6)	64 (65.3)	
Puncture site			0.04
Upper calyx	1 (1.9)	5 (5.1)	
Middle calyx	7 (13.2)	3 (3.1)	
Lower calyx	45 (84.9)	90 (91.8)	

Values are presented as mean ± standard deviation or number (%)

*BMI* Body mass index

Preoperative patient characteristics, except stone type, multiplicity, and puncture site, did not differ significantly between the two groups. However, more complete staghorn stones were observed in the KMP group, whereas more cases of multiple stones were observed in the PCN group. Lower calyx punctures were performed more frequently in the KMP group (Table 1).

Table 2 shows the comparison of the surgical outcomes between PCN and KMP groups. Most surgical outcomes, such as operation time, stone-free rate, post-operative hospitalization period, and complication rate, were comparable, while RET was significantly longer in the PCN group (PCN: 43.77 ± 21.94 vs. KMP: 18.19 ± 10.73; *p* < 0.001). Postoperative complications were classified as febrile urinary tract infection, blood transfusion, and angioembolization due to pseudoaneurysm or continuous bleeding. Of the 151 patients, 6 (4.0%) underwent angioembolization, 11 (7.3%) received blood transfusion, and 18 (11.9%) had febrile urinary tract infection; however, there was no significant difference in the incidence of postoperative complications between both groups.

**Table 2:** Comparison of surgical outcomes between PCN and KMP groups

Variable	PCN (N = 53)	KMP (n = 98)	*P* value
Hospital stay (day)	9.38 ± 4.31	8.95 ± 3.52	0.511
Stone free rate (%)	30 (56.6)	50 (51.0)	0.879
Grade A	19 (63.3)	34 (68)	
Grade B	6 (20)	8 (16)	
Grade C	5 (16.7)	8 (16)	
OP time (min)	82.3 ± 40.21	74.55 ± 30.64	0.188
Rad exposure time (sec)	43.77 ± 21.94	18.19 ± 10.73	< 0.001
Hb drop (g/dL)	1.52 ± 1.24	1.77 ± 1.38	0.263
Complication			0.179
None	47 (88.7)	71 (72.4)	
Angioembolization	1 (1.9)	5 (5.1)	
Transfusion	2 (3.8)	9 (9.2)	
Febrile UTI	3 (5.7)	15 (15.3)	

Values are presented as mean ± standard deviation or number (%)

*Hb* Hemoglobin

We analyzed how the differences between the two groups affect RET, renal stone type, multiplicity of renal stones, and puncture site, which were significantly different between the two groups, were corrected, and performed multivariate regression analysis for catheter type. Table 3 shows the regression coefficient; it was 25.633, which reduced the RET by 25.633 s in the KMP group compared to the PCN group.

**Table 3:** Multivariate regression analysis for RET as a dependent variable

	Unstandardized coefficient		Standardized coefficient	t	*P* value
	B	Standardization error	β		
Constant	72.601	11.361		6.390	0.000
Catheter type	− 25.633	2.800	− 0.621	− 9.156	0.000
Renal stone type	0.417	1.907	0.015	0.219	0.827
Multiplicity of renal stone	− 1.199	2.635	− 0.030	− 0.455	0.650
Puncture site	− 0.785	2.853	− 0.018	− 0.275	0.784

Dependent variable: Radiation exposure time (s)

## Discussion

Several positions have been introduced to overcome the shortcomings of the existing prone position, such as the prone split-leg position [11], reverse lithotomy position [12], lateral decubitus position [13], and supine position [5]. The GMSV position was designed to implement endoscopic combined intrarenal surgery (ECIRS), which allows both antegrade and retrograde approaches [14]. Since its introduction, GMSV position has gained significant popularity. However, so far, none of the randomized clinical trials have compared the results of each position, although postures appropriate for a given surgical method have been applied.

If the modified tract dilation technique is applied, tract dilation and Amplatz sheath placement can be easily achieved. In this technique, the working guidewire should be passed through the bladder. Therefore, if the KMP is placed preoperatively, guidewire passage can be easily achieved with minimal usage of fluoroscopy. However, in some cases, preoperative placement of the KMP can fail because of the presence of complex stones, such as impacted stones in the ureteropelvic junction, large stones in the puncture site or renal pelvis, or staghorn stones. In such cases, a PCN should be placed.

If the KMP is placed preoperatively, guidewire passage can be easily achieved with minimal fluoroscopy. However, if the PCN is placed as a preoperative nephrostomy catheter, several attempts may be needed to properly place the guidewire and tract dilators. This can increase the fluoroscopy usage time and the guidewire may get pulled out. In our study, the mean RET was 18.19 ± 10.73 s in the KMP group and 43.77 ± 21.94 s in the PCN group, showing a statistically significant difference. This result demonstrates that preoperative KMP placement can enable safe and efficient renal tract dilation for PCNL.

Considering that more cases of staghorn stones were observed in the KMP group, the value of the KMP cannot be overlooked.

Table 2 shows the stone free rate seems to be about 50%, which is lower than the results of other reports. However, the criterion for measuring SFR was CT performed at 1 month after surgery. In addition, considering the high rate of staghorn stones (44.4%) and multiple stones in this study, the results were understandable. Previous comparative studies have shown that PCNL performed with a nephrostomy catheter placed before surgery versus access at the time of surgery does not affect the surgical outcomes [15]. Moreover, pre-PCNL nephrostomy can reduce overall operation time, can reduce the rate and severity of postoperative infection, decrease the likelihood of post-PCNL sepsis [7, 8], and radiation exposure for surgeons. In addition, for safe and efficient tract dilation, it is necessary to consider the types of preoperative catheters used for renal access; however, such studies have not yet been reported.

This is the first study to compare the surgical outcomes depending on the type of pre-PCNL catheter used before surgery. Our results showed that the radiation exposure time during PCNL was significantly shorter in the KMP group. Meanwhile, other surgical outcomes, such as stone-free rate, operation time, hospital stay, and complication rates, were comparable between both the groups. Therefore, considering the risk of radiation to surgeons, KMP may be an effective and safe catheter for pre-PCNL nephrostomy, if preoperative renal access is planned.

This study had several limitations. First, as this was a retrospective analysis, the patient groups were not randomly divided. Second, the efficacy and safety of both catheters in other positions, such as the prone position, were not compared. Third, KMP placement as a pre-PCNL nephrostomy is not always possible in all cases. KMP catheter may not be placed in case of impacted stone or some cases of staghorn stone. Fourth, we could not confirm how much radiation was used for pre-operative renal access in the radiology department in this study. Finally, our study reported the experience of our single medical center; therefore, the sample size was relatively small and might not have achieved sufficient power to make a definitive conclusion. Larger population-based prospective randomized studies are required to confirm our conclusions. Nevertheless, this is the first study to compare the efficacy and safety of KMP and PCN as pre-PCNL catheters. The results of this study can guide the future improvements in the surgical techniques for modified supine PCNL.

## Conclusions

Although preoperative placement of KMP in the modified supine position showed comparable stability and effectiveness to that achieved using PCN, it significantly reduced the RET. As the risk of prolonged radiation exposure has been emphasized for patients and surgeons, our findings make valuable contribution to reducing radiation exposure during surgery. Therefore, KMP placement is a safe and effective procedure for performing PCNL in the modified supine position.

## Data Availability

The datasets used and/or analyzed during the current study available from the corresponding author on reasonable request.

## Abbreviations

PCN: Pigtail catheter
KMP: Kumpe access catheter
PCNL: Percutaneous nephrolithotomy
RET: Radiation exposure time
GMSV: Galdakao-modified supine valdivia
UPJ: Ureteropelvic junction
BMI: Body mass index
Hb: Hemoglobin
SFR: Stone-free rate
UTI: Urinary tract infection
WHO: World health organization
ECIRS: Endoscopic combined intrarenal surgery
CT: Computed tomography
